# Editorial: NTM—The New Uber-Bugs

**DOI:** 10.3389/fmicb.2019.01299

**Published:** 2019-06-12

**Authors:** Veronique Dartois, Christine Sizemore, Thomas Dick

**Affiliations:** ^1^Center for Discovery and Innovation, Hackensack Meridian Health, Nutley, NJ, United States; ^2^Division of Microbiology and Infectious Diseases, National Institute of Allergy and Infectious Diseases, National Institutes of Health, Rockville, MD, United States

**Keywords:** non-tuberculous mycobacteria, environmental mycobacteria, lung disease, repositioning, from biology to solutions

Pulmonary disease caused by non-tuberculous mycobacteria (NTM), relatives of *Mycobacterium tuberculosis*, is increasing at an alarming rate, surpassing tuberculosis (TB) in many countries. Patients suffering from chronic pulmonary diseases, including Cystic Fibrosis and Chronic Obstructive Pulmonary Disease, are particularly susceptible to NTM infections. These infections are difficult to diagnose and present significant challenges for treatment. The environmental reservoir of the infectious agents and an incomplete understanding of what makes individuals susceptible to infection complicates preventive approaches. Considering the rising incidence and likely underreporting of these infections, a clear understanding of the barriers to diagnosis, treatment and prevention, and focused research on the pathobiology and microbiology of NTMs and new methods to combat them is urgently needed.

Recognizing this emerging new health threat and the complex nature of these infections, the National Institute of Allergy and Infectious Diseases organized the first NTM-focused workshop “Advancing Translational Science for Pulmonary NTM Infections” in Rockville, MD, in September 2017. This workshop, bringing together scientists and physicians from the United States and abroad, identified gaps in fundamental biological and epidemiological research and mapped out possible approaches for targeted translational science to expedite improvements in detection and care (Daniel-Wayman et al., 2018). The gathering not only generated increased awareness of this new health issue, it also brought together research disciplines and investigators from across the world who previously had few opportunities to interact, and kick-started new collaborative research activities. Momentum created during this workshop also resulted in the Research Topic “NTM—The New Uber-Bugs.”

NTM basic and translational research are still in their infancy with many questions remaining to understand the biological diversity of the infecting pathogens, their similarities and differences and how these affect pathobiology, transmission, and response to therapy. Furthermore, depending on where NTM infections occur globally, different species of NTM predominate, making rapid diagnostics that can speciate mycobacteria a necessity. This acute need to reduce the biological uncertainties around NTM pulmonary disease by increasing research efforts reminds of the state of knowledge TB research was facing two decades ago. Despite an initial research void in the TB field during the last quarter of the twentieth century, in part driven by the absence of molecular tools and animal models to study this difficult pathogen, significant progress has been achieved since the early 2000s. Knowledge and tools developed for the study of TB disease and approaches and technologies and platforms that have been applied to TB product development can be leveraged for NTM research. For instance, chemical entities with activity against *Mycobacterium tuberculosis* that have been generated in TB screening campaigns can quickly also be tested against NTM and may facilitate repositioning and repurposing of chemical leads and create a complementary approach to de novo drug discovery. By exploiting strategies and tools developed for TB, together with increasing research and product development focused on high-priority NTM species, we believe that significant medical advances for NTM diseases will be achieved in the medium term.

The present Research Topic, “NTM—The New Uber-Bugs” compiles contemporary results and insights from a group of leading researchers into the high-priority research area of NTM infections. It also highlights that in order to truly understand the diverse nature of these infections and their impact on human health, more thorough trans-national collaborations and data sharing will be important. We would like to thank the reviewers for their many thoughtful and insightful comments, and the authors for their excellent contributions. We hope that this collection of manuscripts will stimulate much-needed discussions and research activities on the challenging lung disease caused by the diverse group of pathogens called non-tuberculous mycobacteria.

## Author Contributions

All authors listed have made a substantial, direct and intellectual contribution to the work, and approved it for publication.

### Conflict of Interest Statement

The authors declare that the research was conducted in the absence of any commercial or financial relationships that could be construed as a potential conflict of interest.
